# Reversible Hydrogen Storage Media by g-CN Monolayer Decorated with NLi_4_: A First-Principles Study

**DOI:** 10.3390/nano13040647

**Published:** 2023-02-07

**Authors:** Xihao Chen, Wenjie Hou, Fuqiang Zhai, Jiang Cheng, Shuang Yuan, Yihan Li, Ning Wang, Liang Zhang, Jie Ren

**Affiliations:** 1 State Key Laboratory of Precision Spectroscopy, East China Normal University, Shanghai 200062, China; 2 School of Materials Science and Engineering, Chongqing University of Arts and Sciences, Chongqing 402160, China; 3 Chongqing Key Laboratory of Precision Optics, Chongqing Institute of East China Normal University, Chongqing 401120, China; 4 School of Computer Science and Technology, Northwestern Polytechnical University, Xian 710129, China; 5 School of Science, Key Laboratory of High Performance Scientific Computation, Xihua University, Chengdu 610039, China; 6 School of Electric and Electrical Engineering, Shangqiu Normal University, Shangqiu 476000, China; 7 Material Science and Engineering Department, City University of Hongkong, Hongkong 999077, China

**Keywords:** reversible hydrogen storage, DFT, NLi_4_-decorated, g-CN monolayer

## Abstract

A two-dimensional graphene-like carbon nitride (g-CN) monolayer decorated with the superatomic cluster NLi_4_ was studied for reversible hydrogen storage by first-principles calculations. Molecular dynamics simulations show that the g-CN monolayer has good thermal stability at room temperature. The NLi_4_ is firmly anchored on the g-CN monolayer with a binding energy of −6.35 eV. Electronic charges are transferred from the Li atoms of NLi_4_ to the g-CN monolayer, mainly due to the hybridization of Li(2s), C(2p), and N(2p) orbitals. Consequently, a spatial local electrostatic field is formed around NLi_4_, leading to polarization of the adsorbed hydrogen molecules and further enhancing the electrostatic interactions between the Li atoms and hydrogen. Each NLi_4_ can adsorb nine hydrogen molecules with average adsorption energies between −0.152 eV/H_2_ and −0.237 eV/H_2_. This range is within the reversible hydrogen storage energy window. Moreover, the highest achieved gravimetric capacity is up to 9.2 wt%, which is superior to the 5.5 wt% target set by the U.S. Department of Energy. This study shows that g-CN monolayers decorated with NLi_4_ are a good candidate for reversible hydrogen storage.

## 1. Introduction

Hydrogen energy is a promising alternative to traditional fossil fuels due to its highest energy density and the environmental friendliness of its combustion products. Therefore, interest in hydrogen storage is growing due to increasing environmental protection requirements and the trend of low-carbon development. It is expected that the demand in the global hydrogen storage market will expand at a rate of 5.8% from 2019 to 2025 [1]. However, there is a gap between this demand and existing storage technology, where materials play a critical role.

Conventional strategies such as high-pressure compression and liquefaction suffer from low safety and high cost [2]. Therefore, solid-state hydrogen storage is becoming an attractive alternative. A suitable storage medium should have good reversibility between adsorption and desorption and an adequate binding energy of about −0.1 to −0.2 eV per H_2_ [3]. The energy requirement for reversible adsorption just falls into the range of physical adsorption, which guarantees reversible and fast dynamics. Motivated by this consideration, researchers have focused on various nanostructured materials, including two-dimensional (2D) sheets (boron-based nanomaterials, graphene-like nanomaterials, MXenes, MoS_2_, CxNy, etc.) [4,5,6,7,8,9,10,11,12,13], metal atom-modified covalent organic frameworks (COFs) [14,15,16], and metal organic frameworks (MOFs) [17,18,19,20,21]. These materials can exhibit significantly improved kinetics during the adsorption process by decreasing the enthalpy of formation and hydrogenation temperature, and they exhibit many other catalytic effects [22,23,24].

Two-dimensional materials are believed to provide a pathway for the design of next-generation hydrogen storage media due to their superior physical and chemical properties, including their large surface-to-volume ratios, abundant active sites, light weight, and adjustability [25]. However, it is challenging for 2D materials to directly adsorb H_2_ molecules because pure 2D materials fail to provide strong electrostatic interaction. The adsorption energies of H_2_ on graphene and BN are only 0.07 eV [26] and 0.03 eV [27], respectively. Therefore, modification and decoration are necessary to improve their storage capacity. Some research has demonstrated that dopants such as alkali metals, alkaline earth metals, transition metals, and functional groups can improve the storage ability of pristine 2D sheets [26,27,28,29,30,31,32,33,34]. Decorating Mg [28], Ti [35], Ca [36], and Li [26] on graphene-like or carbon-based sheets can provide gravimetric capacities in the range of 7.96 wt% to 10.81 wt%, which meet the 5.5 wt% target value proposed by the U.S. Department of Energy (DOE) in 2020. In addition to single particles, superatomic clusters such as NLi_4_ are also an eye-catching option. The suitability of these clusters has been confirmed by time-of-flight powder neutron diffraction experiments [37,38]. Like alkali metal ions, NLi_4_ has low ionization potentials, which means that decorated NLi_4_ can easily transfer electrons to the substrate and become positively charged. Consequently, an enhanced local electrostatic field can be obtained, which is beneficial for improving hydrogen storage capacity compared to the use of single particles. However, unlike their alkali metal counterparts, the robust binding of Li-N provides superior stability for these clusters. This guarantees a stable connection between the clusters and substrate in addition to less aggregation between the clusters. Some research has provided theoretical support for the significant potential of NLi_4_ for hydrogen storage [33,38,39]. Xiang Wang et al. found that NLi_4_ can enhance the adsorption performance of boron-based 2D materials by forming covalent bonds between the N atoms of NLi_4_ clusters and the B atoms of an h-BN sheet. The average adsorption energy per H_2_ is around −0.20 eV, and the capacity can reach 9.40 wt% [40]. NLi_4_ clusters show better performance when decorated on graphene-like substrates with better conductivity. Hao Qi et al. reported that NLi_4_-decorated graphene can achieve a hydrogen storage capacity of 10.75 wt% with an average adsorption energy of −0.21 eV/H_2_ due to its ideal adsorption strength and abundance of anchor sites [33].

As a type of 2D material, the graphene-like g-CN monolayer structure is composed of uniform holes and aromatic benzene rings containing three N atoms and three C atoms alternatively arranged in the ring [26]. This material is a member of the CxNy family, which is a group of heteroatom-doped carbon materials. A typical and well-known CxNy is g-C_3_N_4_, whose unique structure makes it an attractive candidate for both photocatalytic and electrochemical applications [41]. Many studies have verified the availability and structure adjustability of C_3_N_4_ by various methods, including physical and chemical vapor deposition, thermal condensation, microwave-assisted processes, electrodeposition, hydrothermal and solvothermal synthesis, and sol-gel processes [42,43,44]. In addition to g-C_3_N_4_, the synthesis strategies for preparing of CxNy materials with other ratios, such as C_3_N_5_, C_3_N_3_, C_2_N, and CN, have been developed in the past decade [45]. The substrate materials in our designed CN can be obtained by reacting C_3_N_3_Cl_3_ with molten alkali Na and K, indicating its potential for practical use [46]. G-CN is a semiconductor whose bandgap can be adjusted by dopants [41], and it is expected to have superior hydrogen storage capacity due to its large surface-to-volume ratio and porous geometry structure, which provide sufficient adsorption sites for H_2_ molecules. Some theoretical studies have demonstrated that the decoration of Li [26], Mg [47], and Al [48] noticeably enhances gravimetric density from 6.5 wt% to 10.81 wt%, and the average adsorption energy of H_2_ molecules is in the range of −0.1 to −0.23 eV.

In this work, we used first-principles calculations to design a novel material consisting of NLi_4_ clusters decorated on a g-CN monolayer for hydrogen storage applications. The main motivations for the decoration of NLi_4_ are: (i) unlike general metal ions, the NLi_4_ superatomic cluster can alleviate the cluster effect and can be evenly distributed on the 2D substrate; (ii) NLi_4_ is firmly adsorbed on the surface of the g-CN monolayer (the binding energy is −6.35 eV, which is lower than the previously reported −6.178 and −2.47 eV) [33,38]; (iii) both dopants (NLi_4_ and g-CN) only contain superlight elements, which means that their complex has higher gravimetric capacity for hydrogen storage; (iv) g-CN is favorable for excellent hydrogen storage performance due to its flexible adjustable electronic structure and porous geometry. The average adsorption energy per H_2_ of the NLi_4_@g-CN is between −0.152 eV and −0.237 eV, and a high gravimetric density of 9.2 wt% can be achieved, which considerably surpasses the DOE target value of 5.5 wt%. In addition, the partial density of states (PDOS), charge density, Bader charge analysis, adsorption process, and storage mechanism were thoroughly investigated, and the results also support the excellent hydrogen storage performance of NLi_4_@g-CN. We hope our design and analysis will lay a foundation for the further practical application of advanced energy materials.

## 2. Computational Details

All calculations were conducted by the Vienna Ab initio Simulation Package (VASP) using the generalized gradient approximation (GGA) with the Perdew–Burke–Ernzerhof (PBE) functional under periodic boundary conditions [49,50]. The core electron interactions were described via the projector augmented wave (PAW) approach [51], and the electronic states were approximated by the solution of the plane waves. To avoid coupling effects among the periodic structures, a 20 Å vacuum layer was added on the slab model along the direction perpendicular to the sheet plane. The expansion cut-off energy of the plane wave was set to 520 eV. The optimized structures were obtained by relaxation with a conjugated-gradient algorithm, where the energy convergence of the atomic position and lattice parameters was 1 × 10^−5^ eV. The Hellmann–Feynman forces on each atom were converged within 0.02 eV/Å. The spin effects were considered, and van der Waals corrections were also applied via the DFT-D2 method of Grimme. A Brillouin zone was set for the cell with Gamma-centered k-point grids of 3 × 3 × 1 and 20 × 20 × 1 (based on the convergence tests shown in Supplementary Materials) for structural optimization and partial density of states (PDOS) calculations, respectively [50,52]. The charge transfer between the NLi_4_ superatomic cluster and g-CN was described by Bader charge analysis [53,54].

## 3. Results and Discussion

**Figure 1:**
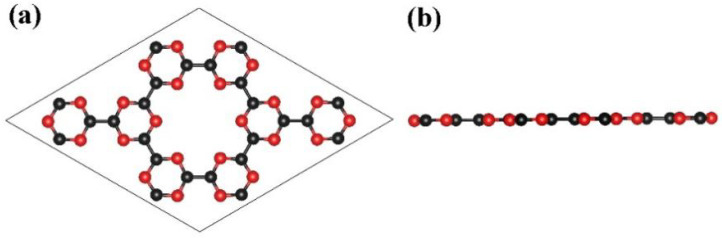
(**a**) Top view and (**b**) side view of the optimized structure of g-CN monolayer. Red and black balls represent N and C atoms, respectively.

**Figure 2:**
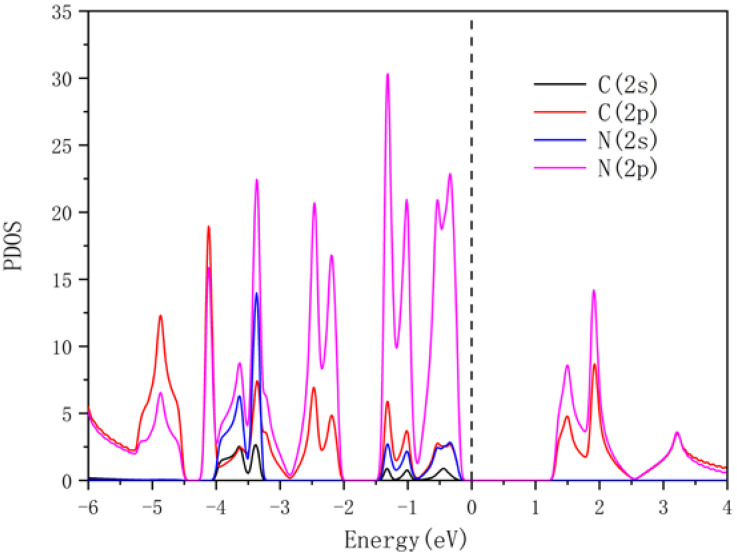
The PDOS of pure g-CN monolayer. The PDOS is shown for carbon and nitrogen atoms. Energy calculation was conducted with reference to Fermi energy level.

**Figure 3:**
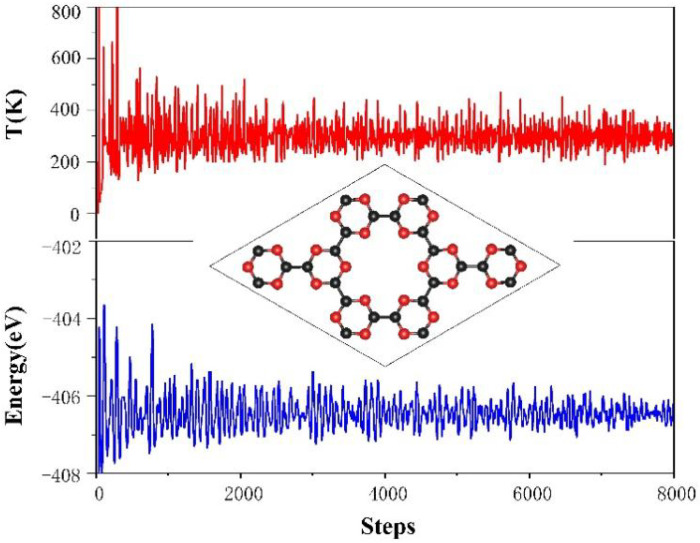
The first-principles MD study about g-CN monolayer. Temperature (**red**) and energy (**blue**) under room temperature (300 k) against time. The time step is 0.5 fs and the total testing is 4 ps, including 8000 steps.

In this work, the optimized lattice parameters of the 2 × 2 × 1 g-CN supercell are a = b = 1.424 nm, and its structure is shown in Figure 1. The g-CN supercell consists of C-N (CN) 6-membered rings and CN 18-membered rings formed by the connections among the CN 6-membered rings, which is identical to previous reports [26]. The porosity of the g-CN layer provides sufficient anchoring active sites for the subsequent decoration of NLi_4_ superatomic clusters, indicating great potential for doping and hydrogen storage. The PDOS for the 2s/2p orbitals of the nitrogen and carbon atoms in the g-CN monolayer were calculated, as presented in Figure 2. Hybridizations exist between the C-2p and N-2p orbitals in the energy range of −6 eV to 4 eV, and the valence bands are mainly occupied by C-2p and N-2p. The band gap of g-CN is about 1.3 eV, i.e., the g-CN is a semiconductor. The thermodynamic stability of the g-CN monolayer at room temperature (300 K) was estimated by first-principles molecular dynamics (MD) simulation with the Nose–Hoover thermostat algorithm. As shown in Figure 3, the system energy slightly oscillates around −406.5 eV. Even after exerting thermal perturbation, the original planar structure with the CN 6-membered rings and 18-membered rings is well-retained, reflecting the good thermodynamic stability of the g-CN.

**Figure 4:**
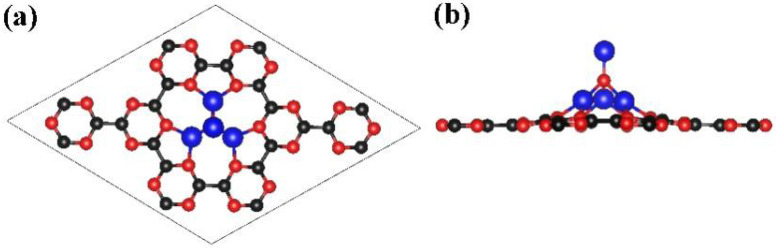
The top view (**a**) and the side view (**b**) of the optimized structure of NLi_4_ decorated on the g-CN monolayer. Red, black, and blue balls are the symbols for N, C, and Li atoms, respectively.

The porous structure of the g-CN monolayer makes it an ideal host for various dopants, including metal atoms and nanoclusters. According to the DFT calculation, the four possible binding sites of NLi_4_ on the g-CN monolayer were tested, including the inner cavity of the large CN 18-membered ring in the center, and the inner cavity of the surrounding small NC rings, C-C bonds, and C-N bonds. It was determined that NLi_4_ can be easily doped on the cavity formed by the large CN 18-membered rings via the formation of ionic bonds between the Li atoms of the NLi_4_ cluster and the N atoms of the g-CN, as shown in Figure 4. The binding energy E_b_ was also calculated according to Formula (1) [55].

(1) $$E_{b}=E_{{\mathrm{NLi}}_{4}\text{@}g-\mathrm{CN}}-E_{{\mathrm{NLi}}_{4}}-E_{g-\mathrm{CN}}$$

where $E_{{\mathrm{NLi}}_{4}\text{@}g-\mathrm{CN}}$, $E_{{\mathrm{NLi}}_{4}}$, and $E_{g-\mathrm{CN}}$ are the energies of the single NLi_4_-decorated g-CN monolayer, an isolated NLi_4_ cluster, and the pure g-CN monolayer, respectively. The binding energy of NLi_4_ and g-CN is −6.35 eV, meaning that a stable interaction exists between NLi_4_ and the g-CN monolayer. The top and side views of the optimized NLi4@g-CN structure (Figure 4) show that g-CN is not twisted by the decoration of NLi_4_. Therefore, the complex still has a high surface-to-volume ratio. The side view shows that the superatomic cluster has a “pyramid building” appearance on the g-CN surface. This is a stable connection and provides enough space for subsequent H_2_ adsorption.

**Figure 5:**
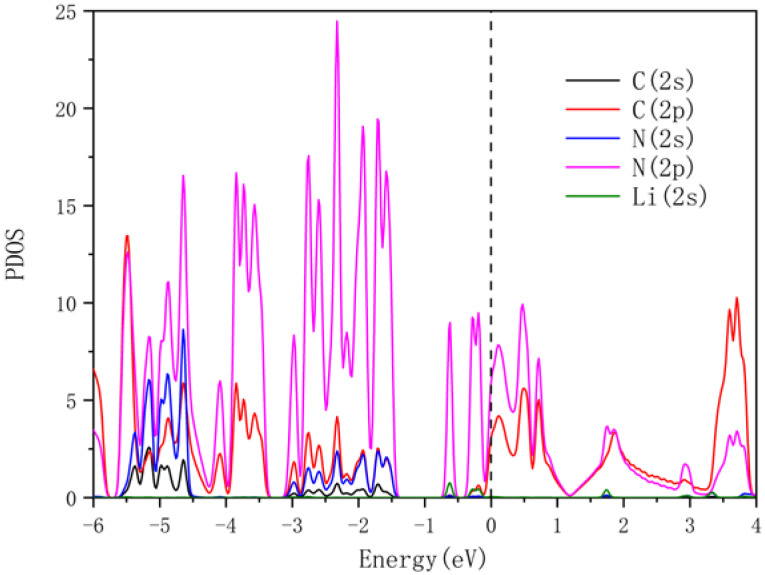
The PDOS of N, C, and Li atoms of NLi_4_ decorated on the g-CN monolayer. Energy calculation was conducted with reference to Fermi energy level.

**Figure 6:**
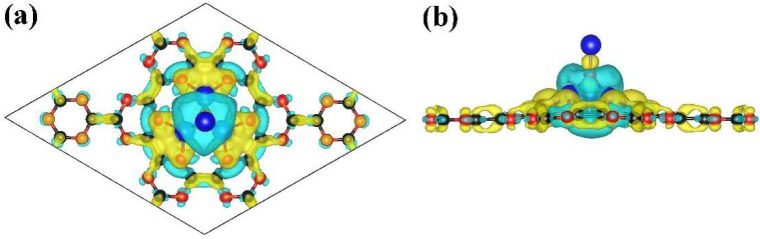
The top view (**a**) and the side view (**b**) of the charge density difference for the NLi_4_ on the g-CN monolayer. Bule and yellow region mean charge loss and gain. The isosurface of charge density is set to 0.0012 (number of charge/Bohr^3^).

The nature of the interaction between the NLi_4_ dopant and g-CN was investigated by PDOS and charge density difference analysis. NLi_4_ decoration can improve the conductivity of g-CN, supporting our assumption that binding formation and charge transfer occur between NLi_4_ and g-CN. Figure 5 shows that the bandgap disappears at the Fermi level, indicating the transformation of the complex from semiconductor to conductor due to the doping of NLi_4_. Clear hybridization between the Li(2s) orbital and the C(2p)/N(2p) orbitals is present at −0.2 and −0.5 eV below the Fermi level, indicating that charge transfer and the formation of ion bonds occur during the combination process. The charge density difference of NLi_4_@g-CN (Figure 6) also provides evidence for this mechanism. There is a clear charge transfer from NLi_4_ to the substrate g-CN. NLi_4_ loses part of its charge, displaying electronegativity. Meanwhile, the g-CN gains charge and displays electropositivity. Thus, a polarization field is formed around NLi_4_, paving the way for subsequent H_2_ adsorption. In addition, the highly charged NLi_4_ superatomic clusters strongly repel each other, which inhibits the aggregation of NLi_4_ molecules. To quantitatively investigate the charge transfer between the dopants and substrate, Bader charge analysis was performed, showing that this transformation is about 0.87 e^−^/atom. This indicates that bonding is formed by strong ionic interaction between the NLi_4_ clusters and g-CN. In other words, a novel material was developed by decorating NLi_4_ on a g-CN monolayer with a large surface-to-volume ratio and favorable electron structure. This is highly promising for achieving excellent H_2_ adsorption performance.

## 4. Hydrogen Adsorption Performance of NLi_4_@g-CN

**Figure 7:**
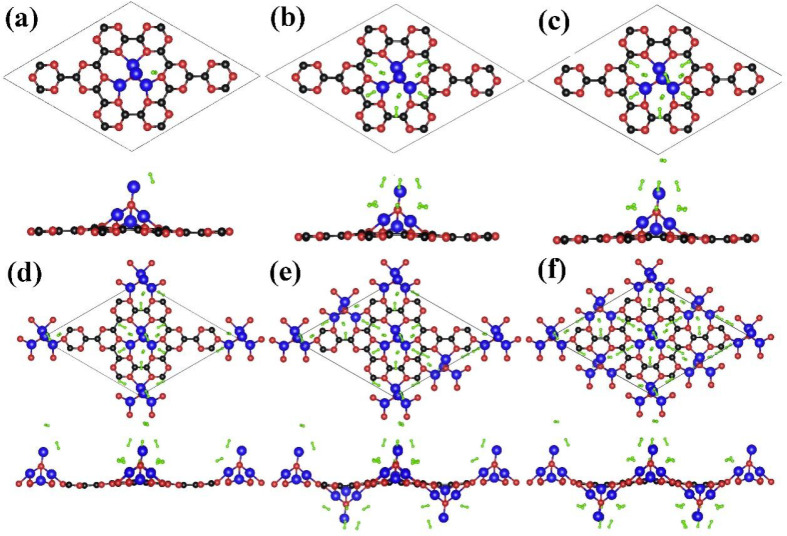
(**a**–**f**) The top and side views of processing of multiple H_2_ absorption on optimized NLi_4_ decorated g-CN monolayer in the increasing order.

**Figure 8:**
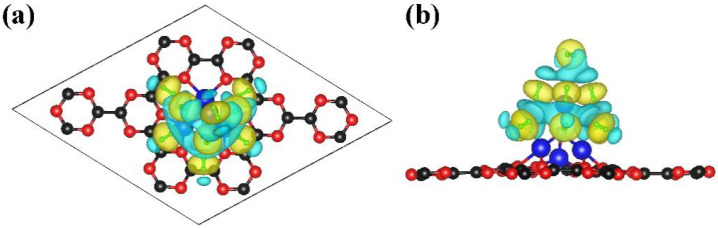
(**a**) Top view and (**b**) side view of charge density difference for the adsorbed H_2_ molecules on NLi_4_-decorated g-CN monolayer, where blue and yellow are charge loss and charge gain region, respectively. The isosurface is set to 0.005 (number of charge/Bohr^3^).

To thoroughly investigate the H_2_ adsorption performance of the NLi_4_@g-CN monolayer, hydrogen molecules were systematically added to the top and side of the Li ions of NLi_4_, followed by structural optimization to obtain the most stable configurations. The corresponding results are shown in the Figure 7. Each NLi_4_ anchored on the g-CN can accommodate a maximum of nine H_2_ molecules. As displayed in Figure 6, the Li ions of the NLi_4_ cluster partially transfer their charge to the substrate, forming a local electrostatic field around the NLi_4_ decoration sites. This is favorable for hydrogen adsorption. This assumption also was confirmed by charge density difference analysis of the NLi_4_@g-CN with adsorbed H_2_, as shown in Figure 8. The notable positive electronic potential around the Li of the NLi_4_ superatomic cluster induces the adsorbed H_2_ to have an unbalanced charge distribution. The center of the H-H bond is a charge-abundant area while the ends are charge-deficient, indicating the polarization of the H_2_ molecules. Therefore, H_2_ is smoothly adsorbed by NLi_4_@g-CN due to the polarization mechanism, and the length of the H-H bond is elongated to 0.76 Å (the bond length of free H_2_ is 0.75 Å [11]). To perform a more quantitative analysis, the average adsorption energy per H_2_ and storage capacity were calculated using Formulas (2) and (3). The average adsorption energy per H_2_ can be written as:

(2) $$E_{\mathrm{ad}}=(E_{{\mathrm{NLi}}_{4}{\text{@}g-\mathrm{CN}-\mathrm{nH}}_{2}}-E_{{\mathrm{NLi}}_{4}\text{@}g-\mathrm{CN}}-nE_{H_{2}})/n$$

where $E_{\mathrm{ad}}$, $E_{{\mathrm{NLi}}_{4}{\text{@}g-\mathrm{CN}-\mathrm{nH}}_{2}}$, $E_{{\mathrm{NLi}}_{4}\text{@}g-\mathrm{CN}}$, $E_{H_{2}}$, and n are the average adsorption energy per H_2_, the energy of the NLi_4_-decorated g-CN monolayer with adsorbed H_2_, the energy of the NLi_4_@g-CN complex, the energy of a single H_2_, and the number of H_2_ molecules, respectively. The storage capacity can be defined as:

(3) $$W_{t}=\frac{n(H)*M(H)}{\left[n(C)*M(C)+n(N)*M(N)+n\left(\mathrm{Li})*M(\mathrm{Li}\right)\right]}$$

where $W_{t}$, n(H), n(C),n(N), and n(Li) denote the storage capacity of the complex and the number of H, C, N, and Li atoms, respectively. M(H), M(C), M(N), and M(Li) denote the molar masses of H, C, N, and Li atoms, respectively. The calculated results are listed in Table 1.

To systematically estimate the conditions for hydrogen desorption from NLi_4_@g-CN, the desorption temperature was calculated by the van’t Hoff equation, which can be written as:

(4) $$T_{d}=\frac{E_{\mathrm{ad}}}{K_{B}}{(\frac{\Delta S}{R}-\mathrm{lnp})}^{-1}$$

where $T_{d}$, $E_{\mathrm{ad}}$, $K_{B}$, ΔS, R and p are the desorption temperature, the average adsorption energy per H_2_, Boltzmann’s constant, the entropy change of H_2_ from gas to solid, the universal gas constant, and the equilibrium pressure, respectively [56]. This work focused on the desorption temperature T_d_ at standard atmospheric pressure, so p = 1 atm and ΔS = 75.44 J K^−1^ mol^−1^ were used for calculation. The estimated desorption temperatures are also listed in Table 1. Table 1 shows that the R_H-H_ value is about 0.76 Å and the T_d_ is between 198–303 K, which is much higher than the critical temperature of H_2_. The average adsorption energy per H_2_ is in the range of −0.152 eV to −0.237 eV, which falls into the range of appropriate reversible adsorption energy. Table 1 also shows that for a single NLi_4_ superatomic cluster, the average adsorption energies per H_2_ are negatively correlated with the number of adsorbed H_2_ molecules, which means that the average adsorption energy per H_2_ decreases with increasing H_2_ loading. This is caused by the repulsion among the adsorbed H_2_, which is in agreement with the behavior reported in Ref. [11]. With an increasing number of NLi_4_ clusters, the adsorption energies per H_2_ maintain proximity as a constant, which means that the storage capacity of g-CN is largely determined by the number of NLi_4_ superatomic clusters. Finally, the highest gravimetric capacity is 9.2 wt%, which surpasses the DOE target of 5.5 wt%.

## 5. Conclusions

In summary, we proposed a promising NLi_4_-decorated g-CN material and estimated its performance for H_2_ storage by first-principles calculations. The NLi_4_ superatomic cluster can be firmly anchored on a g-CN monolayer with a bonding energy of −6.35 eV. The adsorption energies for H_2_ are between −0.152 eV and −0.237 eV, lying within the range of reversible hydrogen storage. Moreover, the storage capacity is as high as 9.2 wt%, which is much higher than the benchmark set by the U.S. DOE. This superior hydrogen storage capacity is attributed to the formation of a spatial local electrostatic field around the NLi_4_ caused by charge transfer from the NLi_4_ superatomic cluster to the g-CN. Hydrogen molecules tend to be polarized due to the formation of this electrostatic field. Thus, electrostatic interaction is enhanced between the hydrogen molecules and the substrate and the adsorption capacity is favorably improved. In addition, the spatial and electronic properties of the NLi_4_ cluster inhibit NLi_4_ aggregation and the repulsion between multiple H_2_ molecules. In the future, we hope that more advanced hydrogen storage materials will be developed along this direction.

## Figures and Tables

**Table 1:** Caption. The average adsorption energy per H_2_, average bond length of H-H, hydrogen storage density and desorption temperature of H_2_ in g-CN system decorated with NLi_4_.

Systems	E_ad_ (eV)	R_H-H_ Length (A)	HSC (wt%)	T_d_ (k)
1NLi_4_@g-CN 1H_2_	−0.237	0.76	0.30	303
1NLi_4_@g-CN 8H_2_	−0.177	0.76	2.42	227
1NLi_4_@g-CN 9H_2_	−0.170	0.76	2.70	217
2NLi_4_@g-CN 18H_2_	−0.155	0.76	5.10	198
3NLi_4_@g-CN 27H_2_	−0.152	0.76	7.30	194
4NLi_4_@g-CN 36H_2_	−0.155	0.76	9.20	198

## Data Availability

Data supporting reported results are available online at https://www.mdpi.com/article/10.3390/nano13040647/s1.

## Supplementary Materials

The following are available online at https://www.mdpi.com/article/10.3390/nano13040647/s1, Figure S1: The convergence tests of total density of states with an increasing k-point grids of N × N × N for pure g-CN monolayer and the optimized structure coordination of pure g-CN monolayer.
