# Supplementary material for: Reversible Hydrogen Storage Media by g-CN Monolayer Decorated with NLi4: A First-Principles Study
Source: Nanomaterials (Basel). 2023 Feb 7;13(4):647. doi: 10.3390/nano13040647 (PMC9964983; doi:10.3390/nano13040647)
Supplement: Supplementary file 1 [file nanomaterials-13-00647-s001.zip › nanomaterials-2158048-supplementary.pdf]

*Supporting information (SI)*

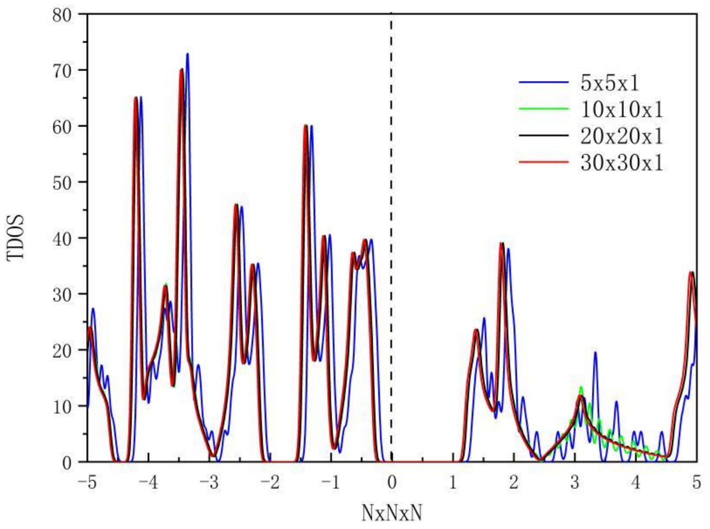


Fig. S1 The convergence tests of total density of states with an increasing k-point grids of N×N×N for pure g-CN monolayer.

The optimized structure coordination of pure g-CN monolayer:

| | | |
|---|---|---|
| 1.00000000000000 | | |
| 14.2329998016000001 | 0.0000000000000000 | 0.0000000000000000 |
| -7.1164999008000001 | 12.3261394002000007 | 0.0000000000000000 |
| 0.0000000000000000 | 0.0000000000000000 | 25.0000000000000000 |
| C | N | |
| 24 | 24 | |

Direct

| | | |
|---|---|---|
| 0.2197116730115027 | 0.2800998494882521 | 0.5008686762526153 |
| 0.2197115434903196 | 0.7800998115366440 | 0.5008686734222962 |
| 0.7197116701909039 | 0.2800998435081468 | 0.5008686679990504 |
| 0.7197115342653433 | 0.7800998163032560 | 0.5008686737948693 |
| 0.2808806973986719 | 0.2189272842200793 | 0.5008738470132101 |
| 0.2808805747539296 | 0.7189272565823899 | 0.5008738440063709 |
| 0.7808807193828358 | 0.2189273439438324 | 0.5008738373976840 |
| 0.7808805848730671 | 0.7189272965227360 | 0.5008738418101260 |
| 0.2196022495707552 | 0.4382849885421436 | 0.5008794060852040 |
| 0.2196021857468224 | 0.9382849670347326 | 0.5008793999394427 |
| 0.7196022109722477 | 0.4382849715442418 | 0.5008794024264347 |
| 0.7196021853584469 | 0.9382850150392272 | 0.5008794061770487 |
| 0.2807695263466385 | 0.0606354622896603 | 0.5008792785952707 |
| 0.2807695311090939 | 0.5606355027063472 | 0.5008792810175891 |

| | | |
|---|---|---|
| 0.7807695468904612 | 0.0606355214792345 | 0.5008792813449574 |
| 0.7807694922896715 | 0.5606355000243823 | 0.5008792812077800 |
| 0.0614209034673934 | 0.2800145027687861 | 0.5008784785916092 |
| 0.0614208064079733 | 0.7800145188641565 | 0.5008784748226134 |
| 0.5614208894607771 | 0.2800144683426211 | 0.5008784790148582 |
| 0.5614208070069253 | 0.7800145874664040 | 0.5008784787106322 |
| 0.4390691237184043 | 0.2188363197214542 | 0.5008771191429560 |
| 0.4390690459615385 | 0.7188364236616209 | 0.5008771195402417 |
| 0.9390691502727719 | 0.2188363829130537 | 0.5008771217033683 |
| 0.9390690456356339 | 0.7188363921963870 | 0.5008771150786018 |
| 0.2786050686979564 | 0.3886892676098057 | 0.5008700020105792 |
| 0.2786049723780479 | 0.8886892162052078 | 0.5008699937629615 |
| 0.7786050360514167 | 0.3886892734653600 | 0.5008699882343919 |
| 0.7786049288062102 | 0.8886892526551051 | 0.5008699986813028 |
| 0.2218751454728789 | 0.1103229410246556 | 0.5008815044008230 |
| 0.2218750653867616 | 0.6103229108770165 | 0.5008815012931009 |
| 0.7218751735597735 | 0.1103230036228666 | 0.5008814925066076 |
| 0.7218750854970111 | 0.6103229537271062 | 0.5008815036398673 |
| 0.1110177055853185 | 0.3886119958957579 | 0.5008848273304238 |
| 0.1110176372718996 | 0.8886120080183275 | 0.5008848212356085 |
| 0.6110176360739459 | 0.3886119671732260 | 0.5008848291827519 |
| 0.6110175955886419 | 0.8886120749107249 | 0.5008848280181653 |
| 0.3893772606606092 | 0.1102479385638375 | 0.5008769563232391 |
| 0.3893772536328015 | 0.6102480522540716 | 0.5008769606334127 |
| 0.8893772735547216 | 0.1102479906292828 | 0.5008769689251409 |
| 0.8893772153328712 | 0.6102480043219387 | 0.5008769608223531 |
| 0.1111198018431523 | 0.2211170994591711 | 0.5008610970804241 |
| 0.1111196782809500 | 0.7211170935232474 | 0.5008610999902388 |
| 0.6111198115304575 | 0.2211170946649545 | 0.5008610985347701 |
| 0.6111196907762491 | 0.7211171371797960 | 0.5008610984292190 |
| 0.3894708151325688 | 0.2778325312195591 | 0.5008641731358665 |
| 0.3894706984795064 | 0.7778325665766488 | 0.5008641716308375 |
| 0.8894708248717649 | 0.2778325889891207 | 0.5008641685924928 |
| 0.8894706910751609 | 0.7778325728305475 | 0.5008641632480320 |

The optimized structure coordination of $NLi_4$ decorated g-CN monolayer with 36 adsorbed hydrogen molecules:

1.00000000000000

| | | |
|---|---|---|
| 14.2329998016000001 | 0.0000000000000000 | 0.0000000000000000 |
| -7.1164999008000001 | 12.3261394003000007 | 0.0000000000000000 |
| 0.0000000000000000 | 0.0000000000000000 | 35.0000000000000000 |

| | | | |
|---|---|---|---|
| C | N | Li | H |
| 24 | 28 | 16 | 72 |

Direct

| | | |
|---|---|---|
| 0.2130840283236869 | 0.2612783452726728 | 0.5031658773518349 |
| 0.2126467238033266 | 0.7607435307093184 | 0.4907121709830309 |
| 0.7127696738241056 | 0.2607982761977834 | 0.4906292320444902 |
| 0.7133877640555996 | 0.7615706450878532 | 0.5037043040427523 |
| 0.2718905164436049 | 0.2012707072390189 | 0.5030573458129781 |
| 0.2725345514750607 | 0.7019474547352575 | 0.4909045196560534 |
| 0.7727345040718865 | 0.2021593389587933 | 0.4901522014797667 |
| 0.7720876450279924 | 0.7017141849768299 | 0.5036095512779784 |
| 0.2092549993894185 | 0.4203425238575929 | 0.4984451877370947 |
| 0.2077302095290377 | 0.9193287731846557 | 0.4953575189323023 |
| 0.7077327539834188 | 0.4191777815425290 | 0.4964395276298517 |
| 0.7094364291567028 | 0.9203359332511376 | 0.4977142397610964 |
| 0.2671850856213867 | 0.0383491214032719 | 0.4963958866531968 |
| 0.2687560686087763 | 0.5394857322240888 | 0.4978711024872889 |
| 0.7690625501110506 | 0.0394859361292846 | 0.4964700959538602 |
| 0.7671932793263980 | 0.5384498369248016 | 0.4974444569980957 |
| 0.0507564017835302 | 0.2576368609307565 | 0.4961145043192154 |
| 0.0495317277589170 | 0.7561322874846974 | 0.4967869574667680 |
| 0.5496929990123367 | 0.2558249429779293 | 0.4975218613007102 |
| 0.5506111910477567 | 0.7577370119644082 | 0.4975298668427494 |
| 0.4304944907756141 | 0.1963259549699709 | 0.4983387329694510 |
| 0.4313908198056390 | 0.6981531277670757 | 0.4967287142998303 |
| 0.9316694462326822 | 0.1981582297406695 | 0.4950016639581858 |
| 0.9304769346637982 | 0.6967968164380626 | 0.4979648898338036 |
| 0.2683779333937351 | 0.3709991464272325 | 0.5049361581186024 |
| 0.2675389732613650 | 0.8703653035895745 | 0.4896015621887090 |
| 0.7673901146326545 | 0.3705705214747373 | 0.4899598708564955 |
| 0.7689395448513539 | 0.8714920853248179 | 0.5040070547519238 |
| 0.2113915390848280 | 0.0912082326263652 | 0.5003618535722129 |
| 0.2128788961616810 | 0.5918421459602948 | 0.4930897240025913 |
| 0.7131044626534342 | 0.0919328171891279 | 0.4922930124190755 |
| 0.7116757312140625 | 0.5912235891259480 | 0.5020129597738473 |
| 0.1009804985626682 | 0.3676030582448503 | 0.4925447778470788 |
| 0.0991640965233485 | 0.8661375996339556 | 0.5001736749002923 |
| 0.5992538079979693 | 0.3656558125972321 | 0.5016886625292111 |
| 0.6006771199119725 | 0.8673779893139488 | 0.4927101068826037 |
| 0.3772288313621158 | 0.0879332698449827 | 0.4930290100197740 |
| 0.3784446912195328 | 0.5897054036325245 | 0.5024306963073780 |
| 0.8789829320361426 | 0.0895832952561943 | 0.5002190272865467 |
| 0.8769904553254984 | 0.5880333283878668 | 0.4932131697477007 |
| 0.1031263151667363 | 0.2015420873126642 | 0.5003664221426659 |
| 0.1024079368884938 | 0.7003068628703903 | 0.4928900422778168 |
| 0.6024890874589991 | 0.2003002921441830 | 0.4929056530832922 |
| 0.6029865200335532 | 0.7019803309680686 | 0.5023915762949899 |

| | | |
|---|---|---|
| 0.3814877393464053 | 0.2558922059742768 | 0.5044493268572131 |
| 0.3823898906697785 | 0.7573536257151627 | 0.4899394624197966 |
| 0.8825717050500348 | 0.2575988033548793 | 0.4888892798117909 |
| 0.8818172125369242 | 0.7566282864564826 | 0.5039262180041092 |
| 0.4986393384774723 | 0.4848670175724967 | 0.5677632903587064 |
| 0.0006508411917807 | 0.9883758810578651 | 0.5654897343943659 |
| 0.4986881355708488 | 0.9886305336954790 | 0.4274336995031073 |
| 0.9984036628067733 | 0.4899793978628773 | 0.4276807080956093 |
| 0.4907743564728265 | 0.3744043107975740 | 0.5366502323264045 |
| 0.5691367414527222 | 0.5589422014708099 | 0.5213764850101937 |
| 0.3855052603667817 | 0.4812437239973927 | 0.5391615345870187 |
| 0.5296746658123970 | 0.4935770386924770 | 0.6175461903875744 |
| 0.9927883030558766 | 0.8748155516189284 | 0.5364199490175641 |
| 0.0641930109981255 | 0.0544275545051289 | 0.5166365898948837 |
| 0.8858372558256453 | 0.9838291458806485 | 0.5377671674078086 |
| 0.0377782039701494 | 0.0013431483907830 | 0.6148897156144718 |
| 0.3861713508259722 | 0.9806289296033779 | 0.4572451789190442 |
| 0.5674453241725246 | 0.0548458268295105 | 0.4756570115987495 |
| 0.4933979734689700 | 0.8745144724979728 | 0.4560674555519257 |
| 0.5125912661574747 | 0.0207892979429388 | 0.3775113162292582 |
| 0.8855427940468474 | 0.4813404692678843 | 0.4577564150762424 |
| 0.0669401613748102 | 0.5543577201435290 | 0.4761419955152030 |
| 0.9942338654579784 | 0.3749354740663992 | 0.4553565965173397 |
| 0.0113156552096828 | 0.5321768244825507 | 0.3787750271616960 |
| 0.5567823671236625 | 0.5110034748964989 | 0.7180026816122453 |
| 0.5247187037123251 | 0.4501980263194594 | 0.7170630365139723 |
| 0.5063133332576560 | 0.3113465722120376 | 0.5846044708042326 |
| 0.5058073334567614 | 0.2614329359729094 | 0.5765292313624094 |
| 0.2794794073134546 | 0.4964782770099690 | 0.5785053910260113 |
| 0.5158708187897522 | 0.6709576503644976 | 0.5774185282797255 |
| 0.5151316854828379 | 0.7226741740395120 | 0.5824802011731226 |
| 0.3283692241516796 | 0.4972189315835410 | 0.5875938947619735 |
| 0.7403888797010684 | 0.5067647681181137 | 0.5799679176911743 |
| 0.3192521877514151 | 0.3090497447982805 | 0.5791108433772623 |
| 0.2653991871632673 | 0.2565343722761622 | 0.5820003980806078 |
| 0.6416726804981318 | 0.4607329224568394 | 0.6341877005045405 |
| 0.6170936734283468 | 0.4586124009049900 | 0.6535459992072417 |
| 0.3946249337719447 | 0.3718947503126473 | 0.6319831595933163 |
| 0.4138006858586108 | 0.3819638382033363 | 0.6526998027649832 |
| 0.5093927088337468 | 0.6108452665219904 | 0.6340270171426537 |
| 0.5173483129593814 | 0.5955241259575900 | 0.6540701815540482 |
| 0.6876519916390316 | 0.5060790348042697 | 0.5752046749824609 |
| 0.0623037356226178 | 0.0137192997637449 | 0.7164836105596396 |
| 0.0292952682588185 | 0.9530007373836030 | 0.7152731184734342 |

| | | |
|---|---|---|
| 0.0115619215402920 | 0.8146916327611394 | 0.5838758148931603 |
| 0.0091790699064192 | 0.7642484263126779 | 0.5753958602081324 |
| 0.7823062395023508 | 0.0027357759266516 | 0.5768611379872106 |
| 0.0178778589266888 | 0.1745094370750819 | 0.5747047281070303 |
| 0.0186340018879612 | 0.2272156162639404 | 0.5795023696467527 |
| 0.8322425168729369 | 0.0054500336388587 | 0.5858939307509740 |
| 0.2426213864106336 | 0.0131851924853470 | 0.5780713009732468 |
| 0.8273896132947897 | 0.8186013821437965 | 0.5781589212258954 |
| 0.7735513854724703 | 0.7661317525598611 | 0.5814643122477621 |
| 0.1488738574034633 | 0.9682949202088106 | 0.6327654976653960 |
| 0.1234750563752050 | 0.9667494939342540 | 0.6518925136595439 |
| 0.9024241234612276 | 0.8826478746258127 | 0.6306719909687292 |
| 0.9227646682449316 | 0.8933448797839032 | 0.6512534346062633 |
| 0.0201624426814046 | 0.1201468729045850 | 0.6313048046496312 |
| 0.0308529973336520 | 0.1068640316664785 | 0.6513099211877902 |
| 0.1887563800507371 | 0.0101442354005172 | 0.5731395183999402 |
| 0.5259612703288129 | 0.0435737176083322 | 0.2760275122541688 |
| 0.4654165662941679 | 0.0094185934427765 | 0.2777058811084055 |
| 0.3247826015334248 | 0.9976622232781643 | 0.4094651967929346 |
| 0.2749880132493696 | 0.9968249126092240 | 0.4178274364188367 |
| 0.5088570843984567 | 0.7672797025799536 | 0.4170643069436143 |
| 0.6828698498207900 | 0.0046995841190933 | 0.4177527859500654 |
| 0.7346977021703327 | 0.0039662231254078 | 0.4127181396835807 |
| 0.5108163449175103 | 0.8165125517890371 | 0.4078022575559609 |
| 0.5196234007546752 | 0.2295377090349973 | 0.4149950191133891 |
| 0.3221778221461638 | 0.8090876916443150 | 0.4152615652798451 |
| 0.2700731338952064 | 0.7550244548131020 | 0.4121406960002963 |
| 0.4798550762144254 | 0.1322928539865397 | 0.3604358579471776 |
| 0.4791783065379148 | 0.1083534183441335 | 0.3410759592419542 |
| 0.3897454502363771 | 0.8862997078131377 | 0.3630166646704268 |
| 0.4005707597789774 | 0.9040819540998352 | 0.3421500102730078 |
| 0.6305336065715402 | 0.0019790141756917 | 0.3602610941665271 |
| 0.6134263748973492 | 0.0094270304621116 | 0.3404684485823719 |
| 0.5188242101572534 | 0.1768113434723318 | 0.4198431222115599 |
| 0.0251528689507431 | 0.5571675325024835 | 0.2763450726873842 |
| 0.9644112309061297 | 0.5233746010512726 | 0.2769197027647922 |
| 0.8234704799319995 | 0.4987698688595004 | 0.4101145954137390 |
| 0.7733076587952556 | 0.4971778546650452 | 0.4184479348059608 |
| 0.0121342239375456 | 0.2729857460804534 | 0.4150600346559979 |
| 0.1855124799075770 | 0.5075100709883270 | 0.4181847428238463 |
| 0.2378374878449500 | 0.5079789308549095 | 0.4131184031417204 |
| 0.0143896923412307 | 0.3231579054087099 | 0.4064583137090191 |
| 0.0252209757488641 | 0.7301399865557736 | 0.4143738653734304 |
| 0.8271415407113243 | 0.3164813606078626 | 0.4151472265353882 |

| | | |
|---|---|---|
| 0.7745074934317596 | 0.2627802933623760 | 0.4120755168366941 |
| 0.9723662176503757 | 0.6389047096400000 | 0.3596384485505940 |
| 0.9701786231072608 | 0.6116108613347344 | 0.3408868276282576 |
| 0.8956901476899315 | 0.3945957640654099 | 0.3631330242066562 |
| 0.9039423954547465 | 0.4143223243112706 | 0.3424689004046442 |
| 0.1293088127504179 | 0.5138835525934042 | 0.3621223508509498 |
| 0.1172487162582820 | 0.5264949426797819 | 0.3422131110558836 |
| 0.0214957936484762 | 0.6762345176779210 | 0.4197760636630585 |
